# Supplementary material for: Hybrid Models and Biological Model Reduction with PyDSTool
Source: PLoS Comput Biol. 2012 Aug 9;8(8):e1002628. doi: 10.1371/journal.pcbi.1002628 (PMC3415397; doi:10.1371/journal.pcbi.1002628)
Supplement: Text S4 — Complete source code for the PyDSTool package (version 0.88.120504). Includes API documentation and help files linking to web pages. This file is identical to the current public release on Sourceforge.net. (ZIP) [file pcbi.1002628.s004.zip › PyDSTool/html/PyDSTool.Toolbox.optimizers.criterion.information_criteria-pysrc.html]

xml version="1.0" encoding="ascii"?


PyDSTool.Toolbox.optimizers.criterion.information\_criteria


| Home | Trees | Indices | Help | PyDSTool |
| --- | --- | --- | --- | --- |

Package PyDSTool ::
Package Toolbox ::
Package optimizers ::
Package criterion ::
Module information\_criteria

[hide private]

[frames] | no frames]

# Source Code for Module PyDSTool.Toolbox.optimizers.criterion.information\_criteria

```
 1   
 2  # Matthieu Brucher 
 3  # Last Change : 2007-08-28 14:42 
 4   
 5  import math 
 6   


7 -class AICCriterion(object):


8    """ 
 9    The Akaike information criterion 
10    """ 


11 -  def __init__(self, ftol, iterations_max, correct_factor = 1.):


12      """ 
13      Initializes the criterion with a max number of iterations and an error fraction for the monotony test 
14      - ftol is the relative tolerance of the AIC criterion 
15      - correct_factor is the modifiying factor for the weight of the parameters size 
16      """ 
17      self.error = ftol 
18      self.iterationMax = iterations_max 
19      self.correct_factor = correct_factor

20   


21 -  def __call__(self, state, **kwargs):


22      """ 
23      Computes the stopping criterion 
24      """ 
25      iteration = state['iteration'] 
26      old_value = state['old_value'] 
27      new_value = state['new_value'] 
28      old_parameters = state['old_parameters'] 
29      new_parameters = state['new_parameters'] 
30      return ((iteration > self.iterationMax) or ((self.correct_factor * len(new_parameters) + new_value) > (self.correct_factor * len(old_parameters) + old_value) * (1. + self.error)))

31   


32 -class ModifiedAICCriterion(object):


33    """ 
34    The Akaike information criterion with several trials authorized 
35    """ 


36 -  def __init__(self, ftol, iterations_max, correct_factor = 1., trials = 5):


37      """ 
38      Initializes the criterion with a max number of iterations and an error fraction for the monotony test 
39      - ftol is the relative tolerance of the AIC criterion 
40      - trials indicates how many time the criterion will return false when in fact the criterion was true 
41      - correct_factor is the modifiying factor for the weight of the parameters size 
42      """ 
43      self.error = ftol 
44      self.iterationMax = iterations_max 
45      self.trials = trials 
46      self.correct_factor = correct_factor

47   


48 -  def __call__(self, state, **kwargs):


49      """ 
50      Computes the stopping criterion 
51      """ 
52      iteration = state['iteration'] 
53      old_value = state['old_value'] 
54      new_value = state['new_value'] 
55      old_parameters = state['old_parameters'] 
56      new_parameters = state['new_parameters'] 
57      if not 'trial' in state: 
58        state['trial']=0 
59      criteria = ((self.correct_factor * len(new_parameters) + new_value) > (self.correct_factor * len(old_parameters) + old_value) * (1. + self.error)) 
60      if criteria: 
61        state['trial'] += 1 
62      return ((iteration > self.iterationMax) or (criteria and (state['trial'] >= self.trials)))

63
```

  


| Home | Trees | Indices | Help | PyDSTool |
| --- | --- | --- | --- | --- |

|  |  |
| --- | --- |
| Generated by Epydoc 3.0.1 on Fri May 4 15:24:18 2012 | http://epydoc.sourceforge.net |
